# Inpatient Rehabilitation Outcomes after Primary Severe Haemorrhagic Stroke: A Retrospective Study Comparing Surgical versus Non-Surgical Management

**DOI:** 10.3390/life13081766

**Published:** 2023-08-18

**Authors:** Poo Lee Ong, Justin Desheng Seah, Karen Sui Geok Chua

**Affiliations:** 1 Institute of Rehabilitation Excellence (IREx), Tan Tock Seng Hospital Rehabilitation Centre, Singapore 569766, Singapore; justin_seah@ttsh.com.sg (J.D.S.); karen_chua@ttsh.com.sg (K.S.G.C.); 2 Lee Kong Chian School of Medicine, Nanyang Technological University, Singapore 639798, Singapore; 3 Yong Loo Lin School of Medicine, National University of Singapore, Singapore 119077, Singapore

**Keywords:** rehabilitation, haemorrhagic stroke, decompressive craniectomy, craniotomy, functional independence measure, length of stay

## Abstract

Background: Haemorrhagic stroke, accounting for 10–20% of all strokes, often requires decompressive surgery as a life-saving measure for cases with massive oedema and raised intracranial pressure. This study was conducted to compare the demographics, characteristics and rehabilitation profiles of patients with severe haemorrhagic stroke who were managed surgically versus those who were managed non-surgically. Methods: A single-centre retrospective study of electronic medical records was conducted over a 3-year period from 1 January 2018 to 31 December 2020. The inclusion criteria were first haemorrhagic stroke, age of >18 years and an admission Functional Independence Measure (FIM™) score of 18–40 upon admission to the rehabilitation centre. The primary outcome measure was discharge FIM™. Secondary outcome measures included modified Rankin Scale (mRS), rehabilitation length of stay (RLOS) and complication rates. Results: A total of 107 patients’ records were analysed; 45 (42.1%) received surgical intervention and 62 (57.9%) patients underwent non-surgical management. Surgically managed patients were significantly younger than non-surgical patients, with a mean age of [surgical 53.1 (SD 12) vs. non-surgical 61.6 (SD 12.3), *p* = 0.001]. Admission FIM was significantly lower in the surgical vs. non-surgical group [23.7 (SD6.7) vs. 26.71 (SD 7.4), *p* = 0.031). However, discharge FIM was similar between both groups [surgical 53.91 (SD23.0) vs. non-surgical 57.0 (SD23.6), *p* = 0.625). Similarly, FIM gain (surgical 30.1 (SD 21.1) vs. non-surgical 30.3 (SD 21.1), *p* = 0.094) and RLOS [surgical 56.2 days (SD 21.5) vs. non-surgical 52.0 days (SD 23.4), *p* = 0.134) were not significantly different between groups. The majority of patients were discharged home (surgical 73.3% vs. non-surgical 74.2%, *p* = 0.920) despite a high level of dependency. Conclusions: Our findings suggest that patients with surgically managed haemorrhagic stroke, while older and more dependent on admission to rehabilitation, achieved comparable FIM gains, discharge FIM and discharge home rates after ~8 weeks of rehabilitation. This highlights the importance of rehabilitation, especially for surgically managed haemorrhagic stroke patients.

## 1. Introduction

Stroke is a significant global health concern, imposing a substantial burden on individuals, communities and healthcare systems. It is the second-leading cause of death worldwide, causing 11.6% (10.8–12.2) of total deaths, and the third-leading cause and disability combined [5.7% (5.1–6.2) of total disability-adjusted life years (DALY)] [GBD 2019] [1].

Furthermore, over the past 17 years, there has been a significant increase of 50% in the lifetime risk of developing a stroke. It is now estimated that one in four individuals older than 25 will experience a stroke during their lifetime [2].

Stroke has emerged as the leading cause of disability and vascular-related death in Asia [3]. Asia accommodates over half of the global population, predominantly concentrated in developing countries. Approximately 70% of new stroke incidences and 87% of stroke-related deaths occur in low- and middle-income countries [4,5,6]. Consequently, higher mortality rates associated with stroke were also observed in Asia, surpassing those in Europe, America and Australia [3,7]. Such epidemiological shifts will substantially impact healthcare systems and populations in such resource-limited settings. This underscores the urgent need for effective stroke prevention, hyperacute management, rehabilitation and population-based support systems.

In Singapore, stroke is the fourth-leading cause of death, accounting for ~10 to 12% of all deaths. The crude death rate of stroke is 40.4 per 100,000 individuals [8], contributing to a considerable socioeconomic burden. The crude incidence rate (CIR) in Singapore has also demonstrated a significant increase, rising from 188.9 per 100,000 population in 2010 to 257.6 per 100,000 population in 2019. Despite the rise in the number of stroke episodes, there has not been a corresponding increase in mortality rates [8]. This can be attributed to the timely initiation of stroke treatments in recent years, underscoring the importance of early intervention. Nonetheless, the continued burden of stroke places significant demands on healthcare systems and resources, necessitating ongoing efforts to address prevention, treatment and rehabilitation strategies.

Haemorrhagic stroke (HS) accounts for 10–20% of all strokes and is associated with a 30-day mortality rate of 40% [9], double that of ischemic stroke [10]. Hypertension is the overall largest attributable risk factor for stroke, particularly among South Asian and Chinese populations compared to Caucasian. This partly elucidates the 18% higher incidence of HS within Chinese populations in contrast to Caucasians [11]. South Asians were also found to be younger at the onset of HS (67 vs. 71 years) compared with Caucasian populations [11].

HS is accompanied by a significant burden of disability, with only 20% of HS survivors regaining functional independence, which constitutes double the odds of long-term disability compared to other stroke subtypes [12,13]. Nevertheless, recent data pointed to >40% of severe HS patients achieving good outcomes by 1 year [14] and some studies suggested that HS survivors attained greater functional gains than their ischemic stroke counterparts [15,16].

The main neurosurgical methods for managing severe HS include life-saving decompressive craniectomy and craniotomy [17]. Other methods include placement of intracranial pressure (ICP) monitors, external ventricular drainage (EVD), clot drainage and aspiration or catheter insertions [18]. Comparisons between decompressive craniectomy and craniotomy demonstrated similar 30-day mortality rates and gross functional outcomes based on previous studies [19,20]. Similarly, another multicentre study in Japan comparing decompressive craniectomy and craniotomy found that in-hospital mortality did not differ between the groups, but that the hospital length of stay was longer in patients who underwent decompressive craniectomy [21]. However, a meta-analysis by Zhong Yao et al. [22] found that decompressive craniectomy significantly reduced mortality and might even improve functional outcomes in certain populations.

While previous studies had demonstrated that decompressive craniectomy yields a notable improvement in survival rate, it was also linked with prolonged hospital stays, poorer functional outcome and increased adverse events [23,24,25]. Recent findings, however, suggest that early surgery did not raise morbidity or mortality rates at 6 months and may even offer clinically significant mortality advantages for individuals with spontaneous superficial HS [26]. Further studies revealed that surgical intervention could particularly benefit HS patients with initial GCS scores ranging from 10 to 13, or those with larger HS. Another study by Alam et al., who examined functional outcomes in patients following decompressive craniectomy, found that there were statistically significant but modest improvements in neurological recovery scores over six months. However, overall functional outcomes for these patients were poor, with a modified Barthel index (MBI) below 60 and a modified Rankin Scale (mRS) > 4, implying a moderate-to-severe disability and significant care burdens [23].

Our study was designed to assess and compare the functional outcomes of patients with severe HS who underwent surgical intervention with those who did not, both during admission and upon discharge from rehabilitation. Additionally, our objective was to identify the potential predictors that may exert an influence on the functional outcomes within our study cohort. The surgical intervention group comprised patients who received either acute decompressive craniectomy or acute craniotomy prior to transfer for inpatient rehabilitation. Although there is no universally accepted definition or consensus for the classification of a severe stroke, a commonly used indicator is an early FIM score of 18 to 40, as indicated by the Evidence-Based Review of Stroke Rehabilitation (EBRSR) [27].

## 2. Materials and Methods

### 2.1. Study Design

A single-centre retrospective review of inpatient electronic medical records was conducted over 3 years from January 2018 to December 2020 in the tertiary rehabilitation unit at Tan Tock Seng Hospital Rehabilitation Centre. The demographics, stroke characteristics and functional data were independently extracted from electronic medical records. This study was approved by the National Healthcare Group-NHG Doman Specific Review Board (NHG DSRB 2021/00132) prior to data extraction. Informed consent was waived by NHG-DSRB as no personal identifiers nor human subjects were involved.

### 2.2. Study Settings

The study was conducted at Tan Tock Seng Hospital (TTSH, Singapore) Rehabilitation Centre, Singapore, which is directly affiliated with National Neuroscience Institute’s (NNI, Singapore) acute stroke unit and neurosurgical unit as a level II trauma centre. Eligible stroke patients were admitted to TTSH rehabilitation centre for further inpatient rehabilitation after undergoing initial screenings by rehabilitation physicians.

The stroke rehabilitation programme comprised multidisciplinary rehabilitation therapies led by consultant rehabilitation physicians with therapies 3 h daily, 5 days a week consisting of physiotherapy, occupational therapy, and speech therapy for an hour each. The therapist-to-patient ratio was 1:8. The inpatient rehabilitation programme was modelled after the neurodevelopmental techniques and Bobath principles [28]. Psychological interventions by psychologists and social work interventions were available if indicated. Adjunctive rehabilitation interventions such as robot-aided therapy (Lokomat^®^, Ekso^®^, Armeo^®^ power) virtual reality commercial off-the-shelf (COTS) gaming platforms and neuromuscular electrical stimulation were also performed as clinically indicated by trained therapists.

### 2.3. Study Participants

EMRs were selected based on the following inclusion and exclusion criteria. Inclusion criteria were patients aged > 18 years, first-onset HS confirmed on computed tomography or magnetic resonance imaging with stroke diagnosed by neurosurgeons, admitted to the rehabilitation unit within 180 days of stroke onset and with admission FIM scores of 18 to 40.

Exclusion criteria were EMRs of patients who had suffered ischaemic stroke, subarachnoid haemorrhage, arteriovenous malformations, traumatic brain injury, brain tumours or infections, failure to complete inpatient rehabilitation and incomplete FIM data.

### 2.4. Data Collection Procedures and Study Variables

We identified EMR cases from the bed management unit and functional data from the rehabilitation standing database. Data were extracted without personal identifiers and used to construct a case record form consisting of independent and dependent variables.

Independent variables included for analysis were demographics variables (age, gender, race), presence of comorbidities, Charlson comorbidity index (CCI) [29], acute length of stay (ALOS), site of haemorrhagic stroke, Glasgow coma scale (GCS) [30], unequal pupils, presence of hydrocephalus confirmed on computed tomography imaging during inpatient stay, intraventricular haemorrhage (IVH), carer needed on discharge and discharge destination. Suitable cases that were complicated with hydrocephalus were treated with ventriculoperitoneal (VP) shunts by a neurosurgeon before transfer to rehabilitation centre.

Dependent variables included for analysis were admission and discharge FIM score, modified Rankin scale (mRS), rehabilitation length of stay (RLOS) and number of complications.

The FIM is an 18-item measure of functional status that comprises motor (13 items) and cognitive (5 items) domains. A motor FIM range of 13–91 and cognitive FIM of 5–35 is obtained. Each individual function is rated from one (total assistance) to seven (complete independence). The total FIM score (sum of motor FIM and cognitive FIM score) can range from a minimum of 18 to a maximum of 126. In this study, a cut-off FIM score of less than 40 points on admission was used to define severe haemorrhagic stroke. Admission and discharge FIM scores were administered by FIM-certified rehabilitation therapists within 72 h of inpatient rehabilitation transfer and prior to discharge from the rehabilitation centre [31].

The modified Rankin Scale (mRS) score was also used to measure patients’ functional status on discharge. It is a seven-point disability scale with scores ranging from 0 (no symptoms) to 6 (dead). It is a commonly used measure that evaluates the level of dependence in activities of daily living for stroke patients [32].

Significant intra-rehabilitation medical complications, defined as those that either needed treatment and/or disrupted rehabilitation, were recorded and managed by the rehabilitation team. For this study, we included the following if they were detected during rehabilitation: post-stroke spasticity, hemiplegic shoulder pain, electrolyte abnormalities, nosocomial infections, neurological or cardiovascular complications and gastrointestinal disorder.

The progress of each patient and the goals set by the multidisciplinary team were monitored and discussed at weekly staff team meetings.

### 2.5. Statistical Analysis

Due to the relatively small sample size, the two surgical interventions of decompression craniectomy and craniotomy were combined into a single group for analysis as the surgically intervened group and then compared with the non-surgical group. There was no significant difference between the two surgical intervention groups in the functional outcome on analysis (*p* = 0.569). The power of the study was 10.2%.

Statistical analyses were generated using SPSS (Statistical Product and Service Solutions) Version 26.0 (IBM Corp., Armonk, NY, USA). Descriptive statistics were used to illustrate patient demographics and stroke characteristics. The Shapiro–Wilk test was used to assess the normality of the data. For normally distributed data, ordinal variables were reported as means with standard deviations (SD), while for non-normally distributed data, medians with lower and upper quartiles (25th, 75th) were used. The comparison of categorical variables was performed using chi-square or Fisher’s exact test. Differences between groups for ordinal data were done using the Mann–Whitney-U test or Kruskal–Wallis test. Correlation tests were run for all variables and only independent variables were selected for linear regression analysis.

A significance level of *p* < 0.05 was considered statistically significant for a two-tailed test.

## 3. Results

### 3.1. Baseline Demographics and Stroke Characteristics

In total, 120 patient EMRs were screened and 13 records were excluded due to incomplete FIM data. The final analysis included a total of 107 eligible patients’ datasets with 45 (42%) patients in the surgical group and 62 (58%) patients in the non-surgical group.

The surgical group comprised patients who underwent either decompressive craniectomy or craniotomy.

Table 1 shows the admission demographic information and stroke characteristics of the cohort. The mean age of the study population was 58.0 years old with the surgical group significantly younger by 8 years (surgical mean 53.1 years vs. non-surgical mean 61.6 years, and *p* = 0.001). The majority of patients were male (64.4%, 69/107) and of Chinese race (73.8%, 79/107). There was no significant difference in gender or racial distribution between the two groups. The prevalence of patients without premorbid comorbidities was found to be 2.6 times higher in the surgical group compared to the non-surgical group (28.9% in the surgical group vs. 11.3% in the non-surgical group, *p* = 0.021).

No significant difference was found between the surgical and non-surgical groups in terms of the site of haemorrhagic stroke (*p* = 0.096). The surgical group had a significantly longer LOS in acute hospital, with an average of 23 days compared to 12 days in the non-surgical group (*p* = 0.000), almost double the duration.

While examining the potential indicators of stroke severity, the surgical group displayed a higher percentage of patients with an initial GCS score of 8 or less (35.6% in the surgical group vs. 8.1% in the non-surgical group, *p* = 0.000). Nonetheless, no significant differences were noted between the groups for other markers such as unequal pupils and intraventricular haemorrhage, except for hydrocephalus (surgical 33.3% vs. non-surgical 11.3%, *p* = 0.005).

### 3.2. Relationship between Surgical Intervention and Functional Outcome Post Stroke Rehabilitation

Table 2 presents the admission and discharge outcomes measured by the FIM score. The admission FIM score was significantly lower in the surgical group compared to the non-surgical group, with a difference of 4 points (*p* = 0.031). However, both discharge FIM score (*p* = 0.625) and FIM gain (*p* = 0.094) displayed similarity between surgical and non-surgical groups.

Likewise, there was no significant difference in the mRS score (*p* = 0.187) between the two groups, indicating similar levels of dependence in activities of daily living. Similarly, the rehabilitation length of stay (RLOS) did not significantly differ between the groups (*p* = 0.134).

Upon discharge from the rehabilitation centre, a substantial majority of 99.1% (106/107) of all patients needed a caregiver, while a significant portion, 73.8% (79/107), returned home after completion of rehabilitation.

### 3.3. Comparison of Number of Complications and Surgical Intervention during Stroke Rehabilitation

Table 3 provides an overview of the number of medical complications encountered by patients across both groups. Our study’s overall complications rate was 91.6%, with the majority (71.0%) suffering two or more complications during their inpatient stay, while the surgical and non-surgical groups showed similar complication rates (surgical 88.9% vs. non-surgical 93.5%, *p* = 0.391). It is worth noting that intra-rehabilitation infections were the most prevalent complication, affecting 60.7% (65/107) of the study population (Supplementary Table S1).

The surgical group did exhibit a relatively higher incidence of central-nervous-system-related complications, encompassing central nervous system infections and seizures, with 13.33% (6/45) of patients in the surgical group compared to 3.23% (2/62) in the non-surgical group. Nevertheless, no significant differences were observed in other sub-categories of medical complications, such as infections, post-stroke pain, spasticity, liver injury and endocrine and cardiovascular complications.

Additional analysis was conducted to explore the potential relationship between the rates of medical complication and functional outcomes. However, our investigation found no significant differences in FIM scores based on the number of complications.

### 3.4. Associations of Stroke Characteristics and Functional Outcomes

We conducted a linear regression analysis to assess the relationship between the total admission FIM score and multiple independent variables. However, the model did not yield significant results. In terms of the total discharge FIM score, we observed a positive correlation with the admission GCS score (*p* = 0.029), while age and RLOS demonstrated negative correlations with discharge FIM score (Table 4).

## 4. Discussion

### 4.1. Group Differences for Demographic and Acute Stroke Characteristics

Our findings highlight several important aspects related to surgical management and rehabilitation outcomes in severe HS patients. Surgically managed HS patients were on average 8 years younger than their non-surgically managed counterparts, albeit slightly younger compared to another local study reporting at 55 years [33]. This finding also corresponds with Taekuchi’s cohort of patients aged 40 to 60 who received surgical intervention [34]. Furthermore, the surgical group of patients demonstrated a lower incidence of comorbidities, potentially related to their relatively younger age.

We observed a higher proportion of patients with an initial GCS score of 8 or less in the surgical group [35.6% (16/45)] compared to the non-surgical group [8.1% (5/62)]. This finding is in line with a previous meta-analysis, indicating that surgery was performed in 30–83% of patients with an initial GCS score of 8 or less [34].

Studies by Kim HT et al. [35] and Akram et al. [36] demonstrated improved survival associated with surgical intervention in HS patients, albeit with an increased length of hospital stay. Our study echoes these results, showing longer acute length of stay in the surgical group (median 23 days in the surgical group vs. 12 days in the non-surgical group), yet comparable complication rates, functional gains and overall outcome between the surgical and non-surgical group despite the differences in length of stay and initial functional status.

### 4.2. Effect of Surgical Intervention and Its Functional Outcomes Post Stroke Rehabilitation

Our study also delved into the relationship between functional outcomes and complications in severe HS patients who underwent surgical intervention and non-surgical management. We found that surgically managed severe HS patients exhibited lower admission FIM scores compared to the non-surgical group. However, there were no significant differences in discharge FIM scores, mRS scores or the FIM gain between the two groups. This suggests that patients receiving surgical intervention for severe HS achieved similar functional benefits as their non-surgical counterparts, without significantly added morbidity during the rehabilitation phase.

Despite the lower admission FIM scores in the surgical group, the ability to attain comparable functional outcomes to the non-surgical group is intriguing. Several factors contribute to this phenomenon. One is the early surgical intervention aimed at reducing pressure in the brain, which will help to reduce brain swelling, increase cerebral blood flow and reduce inflammation [37,38]. Another is the surgical drainage of the haematoma, which also has theoretical advantages such as reduction in intracranial pressure and reduction in excitotoxicity and neurotoxicity of blood products [39,40]. These factors may create a favourable environment for acute neuroplasticity, augmented further by intensive early rehabilitation, leading to improved functional gains [41]. Moreover, the lower FIM score on admission for the surgical group provided greater room for improvement compared to the non-surgical group, potentially explaining the similar FIM gains after rehabilitation. Both groups received intensive rehabilitation during their inpatient stay, which could have played a significant role in improving functional outcome, regardless of their initial FIM scores.

The overall median FIM gain for our study sample was 28 points, which was 9 points higher compared to a study by Ancheta et al. [42] who reported an FIM gain of 19 ± 7 in severe stroke patients after inpatient rehabilitation. The difference in FIM gain could potentially be attributed to our cohort, who were a younger population with a mean age of 58 years, and the longer length of stay (mean 52.7 days). When conducting correlation analysis, we observed a significant negative correlation between age and FIM gain, with a correlation coefficient of −0.266 (*p* = 0.006).

Patients with severe HS demonstrated significant rehabilitation of functional gains, as indicated by a mean total FIM gain of 28 over an average RLOS of 52.7 days. This FIM gain outperformed the threshold for minimal clinically significant difference (MCID) at 22 points and surpassed the FIM gain of 26.1 points observed in stroke patients [43].

### 4.3. Correlational Analysis of Rehabilitation Outcome

Younger age was found to be associated with higher discharge FIM scores (Table 4), corroborating findings from another study that demonstrated younger age’s positive impact on functional outcomes following a stroke [15]. Younger patients may possess higher resilience, robustness, neuroplasticity, cognitive reserve and greater capacity for functional recovery.

Patients with an admission GCS score of 8 or less demonstrated poorer discharge FIM score, consistent with previous studies where surgical intervention did not significantly improve outcomes in HS, with GCS scores of ≤8 [44]. A lower admission GCS score indicates a more severe neurological impairment, and this may impact the potential for functional recovery.

### 4.4. Stroke Rehabilitation Complications

Our study revealed no significant differences in complication rate between the surgical and non-surgical group during their inpatient rehabilitation, when compared with another study [45]. The high complication rates observed in severe stroke patients’ post-surgery could potentially be due to multiple comorbidities, which may increase their vulnerability to complications following surgery. Additionally, the invasive nature of the surgical procedure itself could introduce risk and potential complications.

Moreover, severe stroke patients often have impaired immune systems due to stroke-induced immunosuppression (SIIS) [46], a set of processes that lead to a peripheral suppression of the immune system after the occurrence of a stroke. The combined immune system and neuroinflammation leave stroke patients more vulnerable to infection. In addition, the higher dependency function of severe stroke patient predisposed them to developing thromboembolism, decubitus ulcer and sepsis [47].

From the study, we observed no significant differences in the rates of complications between the surgical and non-surgical groups during inpatient rehabilitation. These findings highlight the significant burden of complications during inpatient rehabilitation for stroke patients, emphasizing the need for comprehensive monitoring and management of potential complications to improve patient outcomes.

Despite the high complication rate, no significant correlation was observed between the high complication rate and FIM gain, possibly due to early medical intervention and ongoing rehabilitation provided during the treatment process.

### 4.5. Post-Rehabilitation Discharge Placement and Carer Status

The majority of our study population (73.8%) were discharged home following inpatient rehabilitation, irrespective of their surgical management, and despite their high care needs. A discharge FIM of 55 and mRS of 4 reflect a moderate level of disability, signifying the need for continuous caregiving after leaving rehabilitation.

This finding aligns with another Asian study that reported a discharge rate of 76.8% for stroke patients after inpatient rehabilitation [48]. The high percentage of patients being able to be discharged home can be attributed to improvement in community resources, Asian close-knit family support systems, ready availability of paid live-in caregivers who are employed as foreign domestic caregivers as well as implementation of early discharge planning [49,50]. These initiatives have facilitated a smoother transition from hospital to home and have provided stroke survivors with the necessary support to continue their recovery in a familiar environment. In Singapore, there are various support groups available, such as the Singapore National Stroke Association (SNSA) [51] and S3 Wellness programmes [52]. These population-based resources play a vital role in providing ongoing assistance and promoting the overall well-being of stroke survivors during their transition back to community living.

### 4.6. Study Limitations

We highlight the following study limitations: The retrospective nature and small sample of our study population may limit the generalizability of the results, in particular to older stroke populations, and hinder the ability to detect significant differences. The mean age of stroke for our study was 58 years, a decade younger than the median age of 69.2 years reported for overall stroke cases in Singapore in 2019 [53].

Furthermore, preselection of patients for rehabilitation may introduce a bias towards a younger population with a higher potential for improvement post-rehabilitation. We also did not collect data on pre-stroke employment, brain imaging details or health-related QOL, which could provide a more comprehensive evaluation of long-term outcomes.

## 5. Conclusions

In conclusion, findings from this study highlight the importance of rehabilitation, particularly in the context of surgically managed stroke patients. While surgically managed haemorrhagic strokes had poorer initial functional outcomes, they achieved comparable discharge functional outcomes to their non-surgically managed counterparts, without discharge or FIM gain differentials. MCID gains were clearly non-inferior to that reported for stroke in general [54]. Nearly all patients needed high amounts of care and a caregiver upon discharge, highlighting the significant societal and economic burden associated with such severe haemorrhagic strokes.

Our study results await further replication, with larger prospective studies comparing rehabilitation outcomes from various surgical interventions such as decompressive craniectomy versus craniotomy, in order to provide valuable insights for functional prognostication, stratification and decisional analysis, in particular for older survivors. Future research could include a long-term follow-up study to determine whether the observed functional gains and discharge outcomes are sustained over time beyond the immediate rehabilitation period.

## Figures and Tables

**Table 1:** Demographics and characteristics of surgical and non-surgical group (n = 107).

Characteristic	Total (n = 107)	Surgical (n = 45)	Non-Surgical (n = 62)	*p*-Value
**Age**				
Years, mean (SD)	58.0 (12.8)	53.1, (12.0)	61.6, (12.3)	**0.001 ^a^**
**Gender, n (%)**				0.409 ^b^
Male	69 (64.4%)	27 (60.0%)	42 (67.7%)	
Female	38 (35.5%)	18 (40.0%)	20 (32.3%)	
**Race, n (%)**				0.841 ^c^
Chinese	79 (73.8%)	32 (71.1%)	47 (75.8%)	
Malay	17 (15.9%)	7 (15.5%)	10 (16.2%)	
Indian	7 (6.5%)	4 (8.9%)	3 (4.8%)	
Others	4 (3.7%)	2 (4.4%)	2 (3.2%)	
**Number of co-morbidities, n (%)**				
0	20, (18.7%)	13, (28.9%)	7, (11.3%)	**0.021 ^b^**
1–2	39, (36.4%)	16, (35.6%)	23, (37.1%)	0.870 ^b^
3–4	48, (44.9%)	16, (35.6%)	32, (52.6%)	0.099 ^b^
**ALOS (days),** median (25th, 75th)	15 (10, 27)	23 (17.5, 35.5)	12 (8, 20.3)	**0.000 ^d^**
**Site of HS, n (%)**				0.096 ^b^
Left	59 (55%)	25 (55.6%)	34 (54.8%)	
Right	40 (37.3%)	17 (37.8%)	23 (37.1%)	
Bilateral	8 (7.5%)	3 (6.6%)	5 (8.0%)	
Markers of stroke severity, n (%) *n = 102				
GCS 8 or less	21 (19.6%)	16 (35.6%)	5 (8.1%)	**0.000 ^b^**
Unequal pupils	8 (7.5%)	5 (22.2%)	3 (4.8%)	0.227 ^c^
Hydrocephalus	22 (20.6%)	15 (33.3%)	7 (11.3%)	**0.005 ^b^**
IVH	37 (34.6%)	20 (44.4%)	17 (27.4%)	0.068 ^b^

^a^ Independent sample *t* test, ^b^ Pearson Chi-Square test, ^c^ Fisher’s exact test, ^d^ Mann–Whitney U test, * missing data. Legends: ALOS: acute length of stay; HS: haemorrhagic stroke; GCS: Glasgow coma scale; IVH: intraventricular haemorrhage.

**Table 2:** Comparison of functional outcome between surgical and non-surgical groups (n = 107).

Variables	Total (n = 107)	Surgical (n= 45)	Non-Surgical (n = 62)	*p*-Value
FIM (admission) Total, median (25th, 75th)	24.0, (18.0, 32.0)	22.0, (18.0, 26.5)	26.0, (18.0, 33.0)	0.031 ^a^
Motor, median (25th, 75th)	15.0, (13.0, 18.0)	13.0, (13.0, 16.5)	15.0, (13.0, 21.3)	0.094 ^a^
Cognition, median (25th, 75th)	7.0, (5.0, 11.0)	5.0, (5.0, 10.0)	8.0, (5.00, 13.0)	0.062 ^a^
FIM (discharge)				
Total, median (25th, 75th)	55.0, (35.0, 74.0)	55.0, (32.5, 73.5)	55.0, (41.0, 75.3)	0.625 ^a^
Motor, median (25th, 75th)	37.0, (25.0, 51.0)	37.0, (22.0, 52.5)	36.0, (24.8, 51.0)	0.960 ^a^
Cognition, median (25th, 75th)	18.0, (11.0, 25.0)	17.0, (8.5, 22.5)	19.0, (12.8, 25.3)	0.228 ^a^
FIM gain				
Total, median (25th, 75th)	28.0, (12.0, 47.0)	28.0, (10.0, 48.0)	26.5, (13.8, 44.8)	0.972 ^a^
Motor, median (25th, 75th)	19.0, (9.0, 33.00)	23.0, (6.0, 36.0)	16.5, (9.75, 30.5)	0.818 ^a^
Cognition, median (25th, 75th)	8.0, (1.0, 15.0)	8.0, (1.0, 14.5)	8.5, (0.0, 15.0)	0.960 ^a^
RLOS (days), mean (SD)	52.7 (23.3)	56.2, (21.5)	50.2, (24.3)	0.187 ^b^
mRS, mean (SD)	4.1, (0.8)	4.1, (0.7)	4.1, (0.9)	0.396 ^c^
Carer needed on discharge				0.262 ^d^
No	1 (0.9%)	0	1 (1.6%)	
Yes	106 (99.1%)	45 (100%)	61 (98.4%)	
Discharge destination				0.920 ^c^
Home	79 (73.8%)	33 (73.3%)	46 (74.2%)	
Others	28 (26.2%)	12 (26.7%)	16 (25.8%)	

^a^ Mann–Whitney U test, ^b^ independent sample t test, ^c^ Pearson Chi-Square test, ^d^ Fisher’s exact test. Legends: FIM: Functional Independence Measure; RLOS: rehab length of stay; mRS: modifed Rankin Scale; IQR: interquartile range.

**Table 3:** Comparison of number of complications between surgical and non-surgical groups.

Number of Complications	Total (n = 107)	Surgical (n = 45)	Non-Surgical (n = 62)	*p*-Value ^a^
0	9 (8.4%)	5 (55.6%)	4 (44.4%)	0.391
1	21 (19.6%)	10 (47.6%)	11 (52.4%)	0.565
1 or more	98 (91.6%)	40 (88.9%)	58 (93.5%)	0.391
2 or more	76 (71.0%)	30 (39.5%)	46 (60.5%)	0.397

^a^ Pearson Chi-Square test.

**Table 4:** Multiple linear regression analysis of stroke characteristics impacting total discharge FIM score (n = 107).

Variables	Multiple Linear Regression		
	Adj. Coeff	95% CI	*p*-Value
Age	−0.330	−1.010, −0.248	**0.001**
Gender	−0.045	−11.781, 7.326	0.644
Race	0.034	−4.800, 6.837	0.729
RLOS	−0.232	−0.439, −0.037	**0.021**
Admission GCS	0.252	3.169, 25.628	**0.013**

Variable selection stepwise method was used; R2 = 0.109. R2 is the percentage of total variance explained by the model. Legends: GCS: Glasgow coma scale; RLOS: rehab length of stay.

## Data Availability

The data presented in this study are available on request from the corresponding author. The data are not publicly available due to institutional review board regulations.

## Supplementary Materials

The following supporting information can be downloaded at: https://www.mdpi.com/article/10.3390/life13081766/s1, Table S1: Types of complications between surgical and non-surgical groups during inpatient rehabilitation.
