# Supplementary material for: Inpatient Rehabilitation Outcomes after Primary Severe Haemorrhagic Stroke: A Retrospective Study Comparing Surgical versus Non-Surgical Management
Source: Life (Basel). 2023 Aug 18;13(8):1766. doi: 10.3390/life13081766 (PMC10455087; doi:10.3390/life13081766)
Supplement: Supplementary file 1 [file life-13-01766-s001.zip › life-2527042-supplementary.pdf]

Table S1. Types of complications between surgical and non-surgical groups during inpatient rehabilitation.

Types of complications, n(%)	Total(n=107)	Surgical(n=45)	Non-surgical (n=62)	P-value[a]
Nosocomial Infection	65(60.7)	27(60)	38(61.3)	0.893
Gastrointestinal bleeding/anaemia	29(27.1)	10(22.2)	19(30.6)	0.333
Mood disorder	25(23.4)	11(24.4)	14(22.6)	0.822
Electrolytes abnormality	20(18.7)	7(15.6)	13(21.0)	0.478
Post stroke spasticity	19(17.8)	8(17.8)	11(17.7)	0.996
Hemiplegic shoulder pain	12(11.2)	4(8.9)	8(12.9)	0.516
Cardiovascular complication	11(10.3)	3(6.7)	8(12.9)	0.294
Neurological complication	8(7.4)	6(13.3)	2(3.2)	**0.050**
Rash	8(7.4)	3(6.7)	5(8.1)	0.786
Liver injury	6(5.6)	4(8.9)	2(3.2)	0.209
Thromboembolism	2(1.9)	0	2(3.2)	0.224
Decubitus ulcer	2(1.9)	2(4.4)	0	0.094

[a] Pearson Chi Square test.
